# Analyzing the Effects of the Kinematic System on the Quality of Holes Drilled in 42CrMo4 + QT Steel

**DOI:** 10.3390/ma14144046

**Published:** 2021-07-20

**Authors:** Mateusz Bronis, Edward Miko, Lukasz Nowakowski

**Affiliations:** Department of Manufacturing Engineering and Metrology, Kielce University of Technology, al. Tysiaclecia Panstwa Polskiego 7, 25-314 Kielce, Poland; emiko@tu.kielce.pl (E.M.); lukasn@tu.kielce.pl (L.N.)

**Keywords:** universal turning center, drilling, kinematic system, ANOVA, hole quality, form errors

## Abstract

This article discusses the relationship between the kinematic system used in drilling and the quality of through-holes. The drilling was done on a CTX Alpha 500 universal turning center using a TiAlN-coated 6.0 mm drill bit with internal cooling, mounted in a driven tool holder. The holes were cut in cylindrical 42CrMo4 + QT steel samples measuring 30 mm in diameter and 30 mm in length. Three types of hole-drilling kinematic systems were considered. The first consisted of a fixed workpiece and a tool performing rotary (primary) and linear motions. In the second system, the workpiece rotated (primary motion) while the tool moved linearly. In the third system, the workpiece and the tool rotated in opposite directions; the tool also moved linearly. The analysis was carried out for four output parameters characterizing the hole quality (i.e., cylindricity, straightness, roundness, and diameter errors). The experiment was designed using the Taguchi approach (orthogonal array). ANOVA multi-factor statistical analysis was used to determine the influence of the input parameters (cutting speed, feed per revolution and type of kinematic system) on the geometrical and dimensional errors of the hole. From the analysis, it is evident that the kinematic system had a significant effect on the hole roundness error.

## 1. Introduction

Drilling is a crucial machining process used in many industrial applications involving hole cutting, which range from those in the tooling sector to those in the machine and space industries [1,2]. A study of the machining operations performed in 145 companies revealed that drilling is the most common machining process in manufacturing [3]. In the case of deep hole cutting, accuracy is essential, as it greatly affects the operation of machines. If holes are drilled incorrectly, for example, in pneumatic control valves, the fluid flow (flow characteristics) may be affected [2]. The hole quality is generally assessed using the following geometrical and dimensional errors: cylindricity, straightness, roundness and diameter [4]. Obtaining high geometrical and dimensional accuracy in drilling is vital as it reduces the production time and, consequently, production costs [5,6].

From a review of the literature, it is clear that the research on drilling has so far focused on the influence of basic process parameters (i.e., cutting speed and feed per revolution) on the hole diameter error [7,8,9,10,11,12]. It can be concluded that the higher the rotational speed of the spindle, the greater the diameter error at the entry and exit of the hole. Unfortunately, research in this field does not include prediction research based on process parameters. There are also no studies showing how clamping errors affect the stability of the drilling process and surface texture of the hole in the case of indexable-insert drills. Such studies have been carried out mainly for milling [5,6].

Some researchers analyzed the effects of number of passes, drill diameter, and coolant pressure on hole quality [1,13,14,15]. They proposed mathematical models for determining hole diameter errors on the basis of the process parameters.

In many cases, research into the influences of the process parameters on the hole form and position errors have involved analyzing a maximum of two output parameters associated with those errors. In the studies described in [11,12], roundness error was measured at the entry and exit of the hole for four values of two process parameters: the rotational speed of the spindle (n = 1000; 3000; 6000; 9000 rpm) and the feed rate ($v_{f}$ = 100; 300; 600; 900 m/min). Some researchers [16,17] have dealt with optimization of the rotational speed of the spindle, feed per revolution, and cutting edge diameter, to improve hole roundness and cylindricity. However, these studies have not presented any general model for predicting the two output parameters on the basis of the input process parameters. Part of the investigations in this area [18,19] has focused on the influence of drill bit coating on hole roundness and straightness as a function of the number of holes drilled. Sandeep et al. [20] analyzed the effects of the rotational speed of the spindle and cooling conditions on the hole roundness. Dheeraj et al. [8] discussed only the influence of the rotational speed of the spindle on hole cylindricity. Zhang et al. [21] attempted to explain the reasons for a hole straightness error observed in 12 tests, and divided the results of admissible hole straightness error into four groups. Denkena et al. [22] studied hole straightness measurement data. The research conducted by Abdelhafeez et al. [23] focused on measuring the straightness and roundness at the entry and exit of the hole for different values of the feed per revolution ($f_{n}$ = 0.24; 0.08 mm/rev.) and cutting speed ($v_{c}$ = 150; 50 m/min). Nevertheless, they did not present any model based on the measurement results. Khanna et al. [24] checked what effect flood cooling or no cooling had on the hole quality (cylindricity and roundness). They did not, however, change any input process parameter. Angelone et al. [9] measured one parameter associated with hole quality (i.e., roundness) for one value of the feed rate and two values of the rotational speed of the spindle (n = 3000; 4500 rpm). Another study [10] consisted of measuring the hole roundness at different values of two input parameters: rotational speed of the spindle (n = 600; 1800; 3000 rpm) and feed per revolution ($f_{n}=$ 0.04; 0.12; 0.2 mm/rev.). In these experiments, two HSS and one sintered carbide drill bits were used. Çiçek et al. [25] developed a model for predicting hole roundness on the basis of three parameters: tool type, cutting speed, and feed per revolution. They concluded that, when combined, the influence of the cutting speed and the feed per revolution on the hole roundness was high, reaching approximately 64%. Aized and Amjad [13] developed models for calculating roundness and cylindricity errors (RE and CE, respectively) in the form of logarithmic equations, taking into consideration the rotational speed of the spindle, the feed rate, and the number of passes. An interesting approach was presented in [14] that involved creating a predictive model to determine hole roundness based on three input parameters: feed rate, rotational speed of the spindle, and coolant pressure. The analysis indicated that the rotational speed of the spindle was one of the most important factors affecting hole diameter (approximately 42%). The investigations described in [24] focused on hole roundness analyzed in relation to feed rate for three different cutting speeds.

From the literature on the subject, it can be concluded that no studies have investigated the relationship between the kinematic system used for drilling and hole diameter, cylindricity, roundness, and straightness errors. Most research so far has considered the effects of the process parameters on hole quality.

The aim of this study was to determine the influence of the process parameters and the type of kinematic system on the holes drilled in 42CrMo4 + QT steel. The experimental data were organized using the Taguchi L27 orthogonal array design, in order to assess the significance of the correlation between the input and output parameters. The computations were performed using a multi-factor ANalysis Of VAriance (ANOVA). The predictive models were developed by applying regression analysis, which is a hybrid method combining polynomial and factorial (fractional) models. The study included simulations based on the predictive models.

## 2. Materials and Methods

The main aim of the study was to analyze the influence of the kinematic system on hole quality in drilling. The experiments were carried out using a DMG MORI CTX alpha 500 universal turning center with a 12-position turret (DMG Mori, Bielefeld, Germany) (direct drive in accordance with VDI 30 DIN 5480) operating at a maximum speed of 5000 rpm, a maximum rotational speed of the spindle of 6000 rpm, a rated power of 20 kW, and a torque of 2200 Nm. Three kinematic systems were considered. The machine design ensured stability of the process.

The drilling was performed using an ATORN HPC UNI 6 mm TiAlNplus solid carbide drill bit (Atorn, Ludwigsburg, Germany) with internal cooling, mounted on a driven tool holder. A TiAlN (titanium aluminum nitride) coating with a microhardness of approximately 30–33 GPa and low thermal conductivity is recommended on tools for machining carbon steel, heat-treated steel, stainless steel, gray iron, aluminum, aluminum alloys, copper, copper alloys, titanium and nickel alloys, and plastics. Table 1 provides the basic parameters of the drill bit used in the experiments.

The material tested was 42CrMo4 + QT steel. This material is very easy to machine and heat treat, but it is not weldable. Characterized by very high ductility and strength, it is commonly used for machine components operating under variable loads. The chemical composition of 40HM+QT steel is shown in Table 2.

Three kinematic systems were used to perform the hole cutting on the universal turning center. The drill bit was clamped in a Sauter VDI 30 (113180) axial drilling head with an ER 25 collet chuck. The drilling was carried out at three different values of the cutting speed ($v_{c}$ = 60, 75, or 90 m/min) and feed per revolution ($f_{n}$ = 0.1, 0.12 or 0.14 mm/rev) to obtain the same depth of cut (a_p_ = 3 mm).

The workpiece was mounted on the spindle in an SMW Autoblok KNCS-N 210-52 self-centering 3-jaw chuck with a gripping force of 6.5 kN per jaw. Adjustable soft jaws were used. Before drilling, the samples were prepared by planing. The through-holes were 6 mm in diameter (l/d = 5).

**Figure 1:**
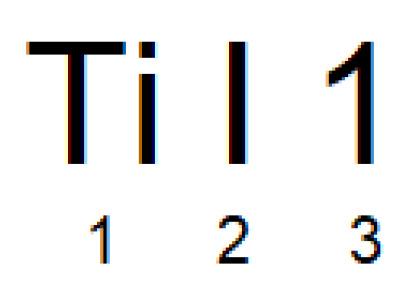
Sample coding.

Figure 1 shows the sample coding method. The first part indicates the type of tool used in the testing, where Ti stands for an ATORN UNI carbide drill bit coated with aluminum titanium nitride. The next element of the code shows the type of kinematic system used (KIN I—the first kinematic system, KIN II—the second kinematic system, and KIN II—the third kinematic system). The last part of the code refers to the set of process parameters.

**Figure 2:**
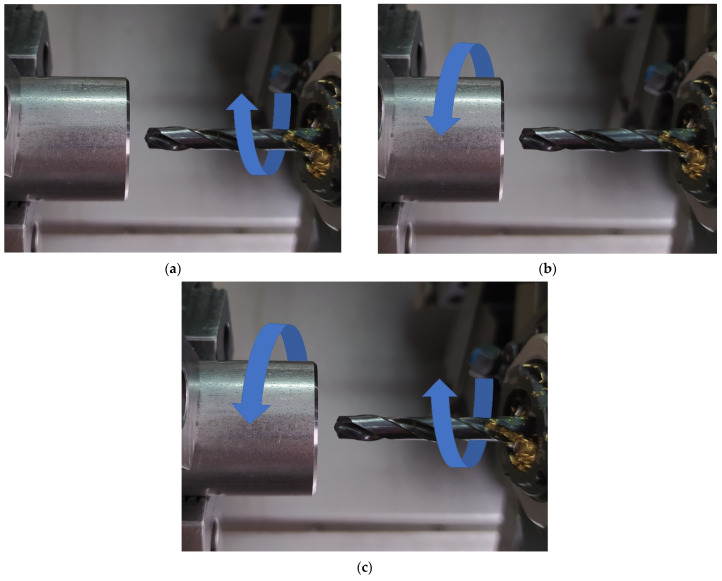
Kinematic systems: (**a**) the first kinematic system, (**b**) the second kinematic system; (**c**) the third kinematic system.

Figure 2 illustrates the kinematic systems used in the hole cutting tests. As can be seen from Figure 2a in the first kinematic system (KIN I), the workpiece is fixed, while the tool performs combined rotary and linear motions (primary and secondary motions). Figure 2b shows the second kinematic system (KIN II) with a rotary workpiece (primary motion) and the tool moving linearly parallel to the workpiece axis of rotation (secondary motion). The third kinematic system (KIN III) shown in Figure 2c has a workpiece and a tool performing rotary motions in opposite directions, with an additional linear motion of the tool (secondary motion).

**Figure 3:**
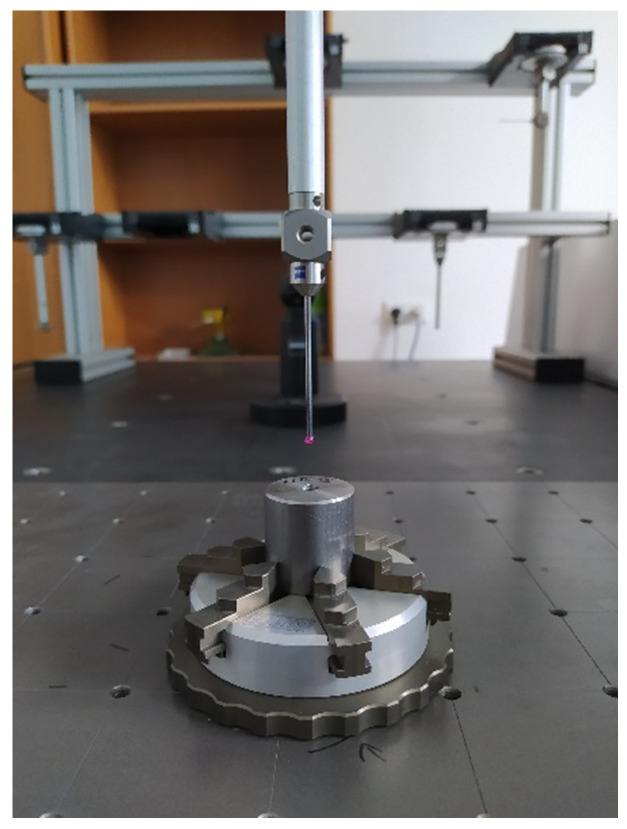
Sample clamping method used in the PRISMO Navigator coordinating machine.

After the drilling, the hole quality parameters (i.e., hole diameter, cylindricity, roundness, and straightness) were determined using a ZEISS PRISMO Navigator coordinate measuring machine (Zeiss, Oberkochen, Germany). The machine selected for the measurements was characterized by high dynamics, high precision, high resistance to ambient conditions, high rigidity, and a high measuring speed. The machine was equipped with an elastomer vibration damping system. The holes were measured using a ruby probe stylus ball tip 3 mm in diameter (Figure 3). The measurements were taken at a speed of 5 mm/s. Each roundness measurement required collecting a total of 1500 points. The same strategy was employed to measure cylindricity (in five cross sections). To assess the roundness and cylindricity errors, a 15 UPR Gaussian filter was used. The straightness error was determined by means of a Gaussian filter with λ_c_ = 2.5 mm. Table 3 shows the basic parameters of the ZEISS PRISMO Navigator coordinating machine parameters.

## 3. Results and Discussions

This section presents the measurement results and analyzes the effects of the input parameters (feed per revolution, cutting speed, and type of kinematic system) on the hole quality (cylindricity, straightness, roundness, and diameter errors).

### 3.1. Design of Experiment and Optimization

The approach proposed by Genichi Taguchi to improve quality and reduce costs in production was used in this study. Table 4 shows the process parameters (cutting speed and feed per revolution) and the type of kinematic system used for each drilling sample.

The L27 Taguchi orthogonal array design was selected to analyze the three independent variables and three level settings for each variable. The number of degrees of freedom was nine.

Table 5 compares the experimental values with the predicted values obtained statistically. The different colors indicate different ranges: green for values below 30%, yellow for 30–70% and red for values above 70%. The range is calculated as the difference between the extreme values obtained with the coordinate measuring machine.

From Table 5, it can be concluded that the most suitable set of parameters is that used for sample TiI1 (*v_c_* = 90 m/min, *f_n_* = 0.14 mm/rev, first kinematic system). For this set, three of the output parameters (i.e., cylindricity, straightness, and diameter errors) were in the lowest range (i.e., below 30%). Most of the predicted values varied 0 ± 2 µm.

### 3.2. Predictive Modelling

There are several types of predictive models (i.e., multiple, polynomial, factorial, and response surface regression models). The response surface model was selected for the analysis because it is a hybrid model combining features of the polynomial and factorial (fractional) regression models. Equation (1) shows the model used to predict two variables.

(1) $$Y=b_{0}+b_{1}X_{1}+b_{2}X_{2}+b_{3}X_{1}{}^{2}+b_{4}X_{2}{}^{2}+b_{5}X_{1}X_{2}$$

where *Y* is the predicted response; $X_{1}$, $X_{2}$ are input process parameters; $b_{0}$ is the free term; and $b_{1}$, $b_{2}$, $b_{3}$, $b_{4}$, $b_{5}$ are coefficients.

### 3.3. Analysis of Variance (ANOVA)

ANOVA is a multi-factor statistical method used to find out if the experimental results are significant. It helps determine the likelihood at which the differences in the mean squares for each observed value are dependent on the analyzed factors. The ANOVA method is suitable for developing a predictive model and checking its significance. A 95% confidence level and 5% significance level were used for the analysis. As can be seen from Table 6, Table 7, Table 8 and Table 9, the results of the ANOVA statistical analysis show how each output parameter is dependent on the input factors.

The values of SS and MS provided in Table 6, Table 7, Table 8 and Table 9 were used to calculate the value of F, which was then checked in the arrays to determine the significance of the statistical analysis. From the analysis, it is apparent that the mathematical models developed for the purpose of this research are significant. The values of *p* are below 0.05, which indicates their significance. The cylindricity error is mainly dependent on parameters $f_{n}$ (21.61%) and $f_{n}{}^{2}\;$(18.52%). Parameters $v_{c}\cdot f_{n}$ (13.49%), $v_{c}$ (11.73%), and $f_{n}$ (9.36%) are reported to have a considerable influence on the straightness error. The roundness error is greatly affected by parameters $v_{c}\cdot KIN$ (30.23%) and KIN (28.04%). Finally, parameters $f_{n}$ (19.20%), $v_{c}$ (14.94%), and $f_{n}{}^{2}$ (14.07%) influence the hole diameter error. The mathematical models based on the empirical observations confirm that the correlation between the input and output variables is high (cylindricity error R^2^ = 69.8%, straightness error R^2^ = 64.17%, roundness error R^2^ = 67.97%, and hole diameter error R^2^ = 72.91%).

The regression model illustrated in Equation (1) was used to analyze the output parameters (i.e., the cylindricity, straightness, roundness, and diameter errors):

(2) $$\begin{array}{c} CYL=212.2407-1.5v_{c}+0.0052v_{c}{}^{2}-23.6722f_{n}+0.8694f_{n}{}^{2}+5.5KIN-0.3556KIN^{2}+0.0406v_{c}\cdot f_{n}\\ +0.0589v_{c}\cdot KIN-0.5958f_{n}\cdot KIN \end{array}$$

(3) $$\begin{array}{c} STR=159.7370-1.7600v_{c}+0.0059v_{c}{}^{2}-13.5458f_{n}+0.3722f_{n}{}^{2}+6.4194KIN-0.7778KIN^{2}+0.0608v_{c}\\ \cdot f_{n}-0.0006v_{c}\cdot KIN-0.1917f_{n}\cdot KIN \end{array}$$

(4) $$\begin{array}{c} RON=-12.5315+0.2526v_{c}-0.0011v_{c}{}^{2}+0.4875f_{n}+0.80028f_{n}{}^{2}+4.0389KIN-0.1389KIN^{2}\\ -0.0036v_{c}\cdot f_{n}-0.0289v_{c}\cdot KIN-0.0917f_{n}\cdot KIN \end{array}$$

(5) $$\begin{array}{c} DE=44.6870-0.4941v_{c}+0.0025v_{c}{}^{2}-4.8264f_{n}+0.1639f_{n}{}^{2}+1.2167KIN+0.2722KIN^{2}+0.0125v_{c}\cdot f_{n}\\ -0.0111v_{c}\cdot KIN-0.0833f_{n}\cdot KIN \end{array}$$

where KIN I = 1; KIN II = 2; and KIN III = 3.

Table 10 shows the average values of the output parameters for each of the three kinematic systems. It is clear that the lowest CYL, STR, and RON errors were observed for the first kinematic system. The CYL and STR errors were the highest for the third kinematic system. For the second kinematic system, the RON error, reaching 4.4 µm, was the highest.

**Figure 4:**
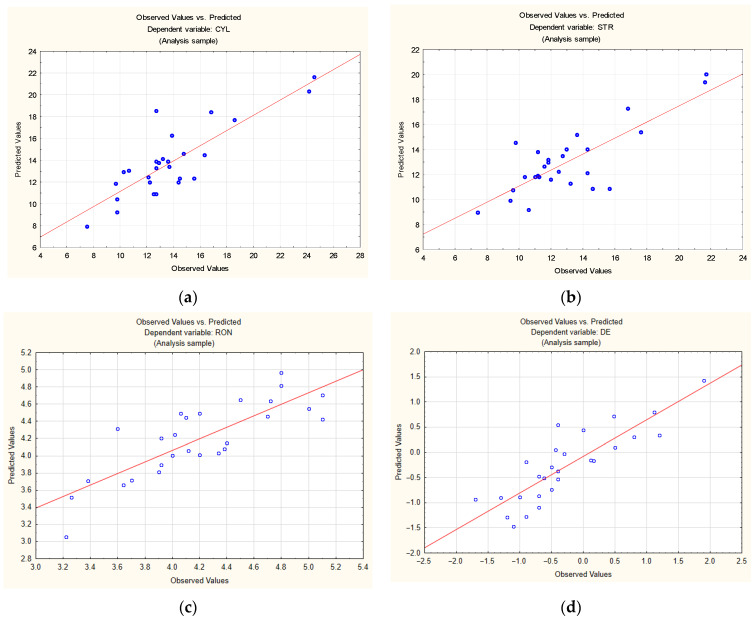
Experimental values vs. predicted values for: (**a**) cylindricity error; (**b**) straightness error; (**c**) roundness error; (**d**) diameter error.

The observed and predicted values of the cylindricity, straightness, roundness, and hole diameter errors provided in Table 5 are compared graphically in Figure 4. As can be seen from Figure 4, the goodness of fit is high (coefficient of determination R^2^ > 60%). The differences between the observed values and the expected values result from the number of experimental runs (27), the large ranges of values of the process parameters and the large differences between the kinematic systems. The goodness of fit would have been higher if the experiment had involved measuring smaller ranges of the process parameters or using only one kinematic system. Whichever the case, errors would have been smaller for a smaller number of factors and level settings.

**Figure 5:**
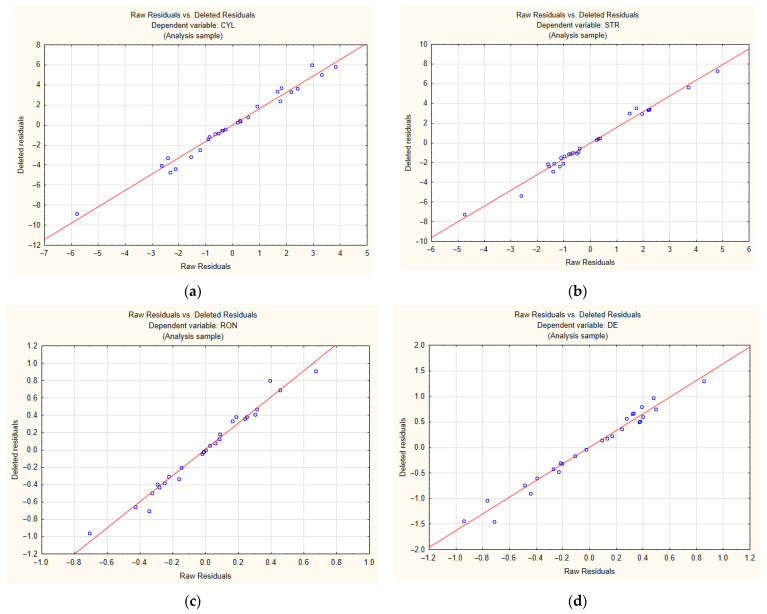
Normal Probability plots for: (**a**) cylindricity error; (**b**) straightness error; (**c**) roundness error; (**d**) diameter error.

Figure 5 shows plots of raw residuals vs. deleted residuals for all the output parameters. From the graphs, it can be concluded that the assumption of normality is fulfilled, as there is a good linear correlation.

### 3.4. Simulations of the Hole Roundness Error

**Figure 6:**
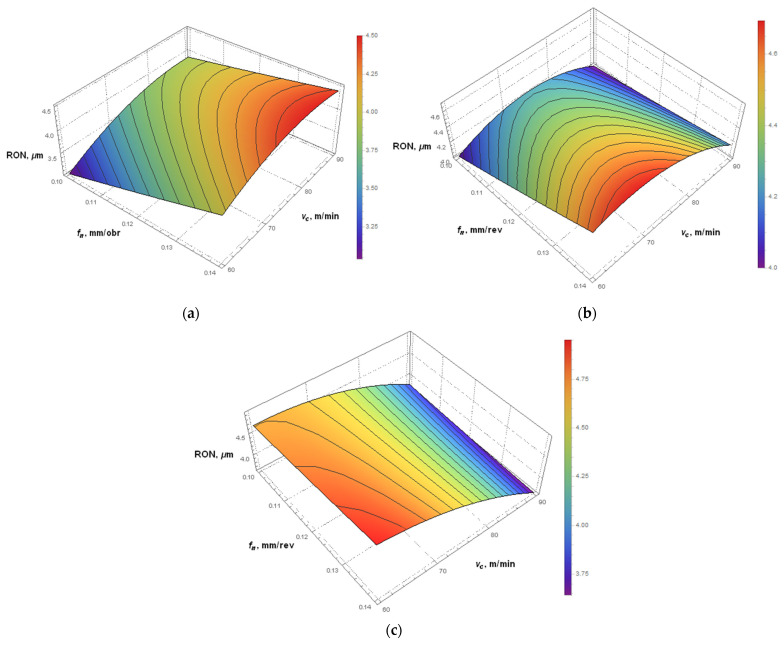
Simulations of the hole roundness error for: (**a**) KIN I, (**b**) KIN II; (**c**) KIN III.

The roundness error was selected for further analysis, as it is greatly dependent on the type of kinematic system used for drilling. The parameters affecting this performance variable are $v_{c}\cdot KIN\;\mathrm{and}\;KIN$. Figure 6a shows that for the first kinematic system, the smallest roundness error was obtained at $v_{c}$ = 60 m/min and $f_{n}$ = 0.10 mm/rev. The higher the parameters, the higher the roundness error. For the second kinematic system (Figure 6b), the smallest roundness error is observed at a low feed per revolution of approximately 0.10 mm/rev and a cutting speed of either 60 or 90 m/min. From the calculations, it is evident that the least favorable conditions are at a high feed per revolution of 0.14 mm/rev and a cutting speed of 70 m/min. For the third kinematic system (Figure 6c), the lowest error (3.6 µm) was obtained at a cutting speed of 90 m/min and a feed per revolution ranging from 0.13 to 0.14 mm/rev. For the third kinematic system, the roundness error increased with decreasing cutting speed. In this case, feed per revolution had very little effect on this hole form error.

## 4. Conclusions

This article analyzed the influences of cutting speed, feed per revolution, and type of kinematic system on the geometrical and dimensional accuracy of holes drilled in 42CrMo4 + QT steel. The study involved developing predictive models for the selected output parameters.

The main conclusions drawn from the study are as follows:

- The empirical mathematical models show a high correlation despite the large number of cases considered; the models may prove useful under industrial conditions when drilling parameters are selected;
- The kinematic system is reported to have a considerable (65.23%) effect on the roundness error of holes drilled in 42CrMo4 + QT steel;
- The Taguchi L27 orthogonal array design can be successfully used to assess the influence of the input process parameters (cutting speed and feed per revolution) and the type of kinematic system on the output parameters (hole cylindricity, straightness, roundness, and diameter errors);
- The kinematic system has a substantial effect on the roundness error—for the third kinematic system, the hole roundness error was high (min. 3.6 µm, max. 5.1 µm); however, for the first kinematic system, its values were small (min = 3.2 µm, max = 4.7 µm);
- The first kinematic system is the most suitable because three out of four output parameters reached the lowest values (CYL = 12.5 µm, STR = 11.3 µm, RON 4.0 µm);
- Future research will focus on the surface texture of holes and burr formation at the exits for different kinematic systems.

## Figures and Tables

**Table 1:** Parameters of the drill bit used.

Specification	
Cutting edge diameter	6 mm
Cutting material	VHM
Coating	TiAlNPlus
Type	HPC UNI
Coolant supply	Internal
Tool holding device	HA parallel shank
Point angle	140°
Shaft diameter	6 mm
Chip flute length	44 mm
DIN	6537

**Table 2:** Chemical composition of 40HM + QT quenched steel.

C	Mn	Si	P	S	Cr	Ni	Mo
0.38–0.45	0.6–0.9	0.1–0.4	Max. 0.035	Max. 0.035	0.9–1.2	Max. 0.3	0.15–0.25

**Table 3:** Parameters of the ZEISS PRISMO Navigator coordinate measuring machine.

Parameter	Value
Measurement range X	900 mm
Measurement range Y	1200 mm
Measurement range Z	700 mm
MPE_E	0.9 + L/350 µm
MPE_P	1.0 µm
MPE_RON_t_	1.0 µm
MPE_THP	1.9 µm

**Table 4:** Input parameters used in the drilling of 42CrMo4 + QT steel.

Experiment No.	Sample Code	*v_c_*, m/min	*f_n_*, mm/rev	KIN
1	TiI1	90	0.14	1
2	TiII1	90	0.14	2
3	TiIII1	90	0.14	3
4	TiI2	75	0.14	1
5	TiII2	75	0.14	2
6	TiIII2	75	0.14	3
7	TiI3	60	0.14	1
8	TiII3	60	0.14	2
9	TiIII3	60	0.14	3
10	TiI4	90	0.12	1
11	TiII4	90	0.12	2
12	TiIII4	90	0.12	3
13	TiI5	75	0.12	1
14	TiII5	75	0.12	2
15	TiIII5	75	0.12	3
16	TiI6	60	0.12	1
17	TiII6	60	0.12	2
18	TiIII6	60	0.12	3
19	TiI7	90	0.1	1
20	TiII7	90	0.1	2
21	TiIII7	90	0.1	3
22	TiI8	75	0.1	1
23	TiII8	75	0.1	2
24	TiIII8	75	0.1	3
25	TiI9	60	0.1	1
26	TiII9	60	0.1	2
27	TiIII9	60	0.1	3

**Table 5:** Experimental vs. predicted values.

	Experimental Results				Predicted Results			
Sample Code	CYL 15 UPR, µm	STR F2.5, µm	RON 15 UPR, µm	DE, µm	CYL 15 UPR, µm	STR F2.5, µm	RON 15 UPR, µm	DE, µm
TiI1	9.7	10.4	4.1	0.0	11.8	11.8	4.4	0.4
TiII1	12.7	11.8	3.9	0.8	13.2	13.2	4.2	0.3
TiIII1	12.7	11.8	3.6	0.5	13.9	13.0	3.6	0.7
TiI2	14.4	15.7	4.7	−1.0	12.0	10.9	4.5	−0.9
TiII2	12.1	12.5	4.5	−0.7	12.5	12.3	4.6	−0.9
TiIII2	15.6	14.3	5.0	−0.5	12.3	12.1	4.5	−0.3
TiI3	16.3	11.6	4.2	−0.7	14.5	12.6	4.0	−1.1
TiII3	13.2	14.3	4.7	−1.3	14.1	14.0	4.6	−0.9
TiIII3	10.7	11.2	4.8	0.2	13.0	13.8	5.0	−0.1
TiI4	7.5	7.4	4.4	−0.6	7.9	9.0	4.2	−0.5
TiII4	9.8	9.6	4.4	−0.7	10.5	10.7	4.1	−0.5
TiIII4	14.5	14.6	3.7	0.5	12.3	10.9	3.7	0.1
TiI5	9.8	9.5	4.1	−1.1	9.2	9.9	4.1	−1.5
TiII5	12.7	12.0	5.1	−0.9	10.9	11.6	4.4	−1.3
TiIII5	12.2	11.0	4.2	−0.4	11.9	11.8	4.5	−0.5
TiI6	10.3	12.7	3.3	−1.2	12.9	13.5	3.5	−1.3
TiII6	12.9	13.6	3.6	−1.7	13.8	15.2	4.3	−0.9
TiIII6	13.6	17.6	4.8	−0.3	13.9	15.4	4.8	0.0
TiI7	12.5	10.6	3.9	0.1	10.8	9.1	3.9	−0.1
TiII7	14.8	13.2	4.0	−0.4	14.6	11.2	4.0	0.1
TiIII7	18.6	11.3	3.9	1.1	17.7	11.8	3.8	0.8
TiI8	13.7	11.2	3.4	−0.5	13.4	11.9	3.7	−0.7
TiII8	13.9	13.0	4.0	−0.4	16.3	14.0	4.2	−0.4
TiIII8	12.7	9.8	4.1	−0.4	18.5	14.6	4.5	0.5
TiI9	16.8	16.8	3.2	−0.9	18.4	17.2	3.0	−0.2
TiII9	24.2	21.6	4.3	1.2	20.4	19.4	4.0	0.3
TiIII9	24.6	21.7	5.1	1.9	21.7	20.0	4.7	1.4

The different colors indicate different ranges: green for values below 30%, yellow for 30–70% and red for values above 70%.

**Table 6:** ANOVA results for the cylindricity error.

Source	SS	DF	MS	*F* Value	*p* Value	Percentage Contribution
Model	273.4319	9	30.3813	4.3652	0.0044	69.80
Constant	110.5175	1	110.5175	15.8793	0.0010	28.21
*v_c_*	25.2424	1	25.2424	3.6269	0.0739	6.44
*v_c_* ^2^	8.3230	1	8.3230	1.1959	0.2894	2.12
*f_n_*	84.6735	1	84.6735	12.1660	0.0028	21.61
*f_n_* ^2^	72.5696	1	72.5696	10.4269	0.0049	18.52
KIN	3.8754	1	3.8754	0.5568	0.4657	0.99
KIN^2^	0.7585	1	0.7585	0.1090	0.7453	0.19
$v_{c}\cdot f_{n}$	17.7633	1	17.7633	2.5523	0.1286	4.53
$v_{c}\cdot KIN$	9.3633	1	9.3633	1.3453	0.2621	2.39
$f_{n}\cdot KIN$	17.0408	1	17.0408	2.4484	0.1361	4.35
Error	118.3177	17	6.9599	-	-	30.20
Total	391.7496	26	-	-	-	100

**Table 7:** ANOVA results for the straightness error.

Source	SS	DF	MS	*F* Value	*p* Value	Percentage Contribution
Model	190.1672	9	21.1297	3.3826	0.0147	64.17
Constant	62.6015	1	62.6015	10.0218	0.0056	21.12
*v_c_*	34.7515	1	34.7515	5.5633	0.0306	11.73
*v_c_* ^2^	10.4896	1	10.4896	1.6793	0.2123	3.54
*f_n_*	27.7256	1	27.7256	4.4385	0.0503	9.36
*f_n_* ^2^	13.3007	1	13.3007	2.1293	0.1627	4.49
KIN	5.2795	1	5.2795	0.8452	0.3708	1.78
KIN^2^	3.6296	1	3.6296	0.5811	0.4563	1.22
$v_{c}\cdot f_{n}$	39.9675	1	39.9675	6.3983	0.0216	13.49
$v_{c}\cdot KIN$	0.0008	1	0.0008	0.0001	0.9909	0.00
$f_{n}\cdot KIN$	1.7633	1	1.7633	0.2823	0.6021	0.60
Error	106.1913	17	6.2465	-	-	35.83
Total	296.3585	26	-	-	-	100

**Table 8:** ANOVA results for the roundness error.

Source	SS	DF	MS	*F* Value	*p* Value	Percentage Contribution
Model	5.0664	9	0.5629	4.0080	0.0067	67.97
Constant	0.3853	1	0.3853	2.7432	0.1160	5.17
*v_c_*	0.7158	1	0.7158	5.0964	0.0374	9.60
*v_c_* ^2^	0.3424	1	0.3424	2.4379	0.1369	4.59
*f_n_*	0.0359	1	0.0359	0.2557	0.6196	0.48
*f_n_* ^2^	0.0007	1	0.0007	0.0053	0.9430	0.01
KIN	2.0899	1	2.0899	14.8796	0.0013	28.04
KIN^2^	0.1157	1	0.1157	0.8241	0.3767	1.55
$v_{c}\cdot f_{n}$	0.1408	1	0.1408	1.0027	0.3307	1.89
$v_{c}\cdot KIN$	2.2533	1	2.2533	16.0434	0.0009	30.23
$f_{n}\cdot KIN$	0.4033	1	0.4033	2.8717	0.1084	5.41
Error	2.3877	17	0.1405	-	-	32.03
Total	7.4541	26	-	-	-	100

**Table 9:** ANOVA results for the diameter error.

Source	SS	DF	MS	*F* Value	*p* Value	Percentage Contribution
Model	13.3653	9	1.4850	5.0831	0.0020	72.91
Constant	4.8993	1	4.8993	16.7698	0.0008	26.73
v_c_	2.7386	1	2.7386	9.3740	0.0071	14.94
v_c_^2^	1.9646	1	1.9646	6.7247	0.0189	10.72
f_n_	3.5198	1	3.5198	12.0478	0.0029	19.20
f_n_^2^	2.5785	1	2.5785	8.8260	0.0086	14.07
KIN	0.1896	1	0.1896	0.6491	0.4316	1.03
KIN^2^	0.4446	1	0.4446	1.5219	0.2341	2.43
$v_{c}\cdot f_{n}$	1.6875	1	1.6875	5.7761	0.0279	9.21
$v_{c}\cdot KIN$	0.3333	1	0.3333	1.1410	0.3004	1.82
$f_{n}\cdot KIN$	0.3333	1	0.3333	1.1410	0.3004	1.82
Error	4.9666	17	0.2922	-	-	27.09
Total	18.3319	26	-	-	-	100

**Table 10:** Average values of the output parameters for each kinematic system studied.

	Kinematic System		
Parameter, Average Value	KIN I	KIN II	KIN III
CYL, µm	12.5	13.8	15.2
STR, µm	11.3	13.2	13.3
RON, µm	4.0	4.4	4.2
DE, µm	−0.7	−0.4	0.3

## Data Availability

Data Sharing is not applicable.

## Nomenclature

DOE	design of experiment
$v_{f}$	feed rate (mm/min)
n	spindle speed (rpm)
$f_{n}$	feed per revolution (mm/rev)
$v_{c}$	cutting speed (m/min)
$a_{p}$	depth of cut (mm)
KIN	kinematics
SS	Sum of Squares
DF	Degrees of Freedom
MS	Mean Square
CYL	CYLindricity deviation (µm)
STR	STRaightness deviation (µm)
RON	ROuNdness deviation (µm)
DE	Diameter Error (µm)
UPR	Undulations per revolution
*F*	wave length
*p*	significance
